# Altered Prefrontal–Basal Ganglia Effective Connectivity in Patients With Poststroke Cognitive Impairment

**DOI:** 10.3389/fneur.2020.577482

**Published:** 2020-12-16

**Authors:** Jing Zhang, Zixiao Li, Xingxing Cao, Lijun Zuo, Wei Wen, Wanlin Zhu, Jiyang Jiang, Jian Cheng, Perminder Sachdev, Tao Liu, Yongjun Wang

**Affiliations:** ^1^School of Biological Science and Medical Engineering, Beihang University, Beijing, China; ^2^Beijing TianTan Hospital, Capital Medical University, Beijing, China; ^3^Centre for Healthy Brain Ageing, School of Psychiatry (CHeBA), University of New South Wales, Sydney, NSW, Australia; ^4^Neuropsychiatric Institute, Prince of Wales Hospital, Sydney, NSW, Australia; ^5^Beijing Advanced Innovation Center for Big Data-Based Precision Medicine, Beijing, China; ^6^Beijing Advanced Innovation Center for Biomedical Engineering, Beijing, China; ^7^China National Clinical Research Center for Neurological Diseases, Beijing, China

**Keywords:** cognitive impairment, prefrontal–basal ganglia circuit, dynamic causal modeling, fMRI, stroke

## Abstract

We investigated the association between poststroke cognitive impairment and a specific effective network connectivity in the prefrontal–basal ganglia circuit. The resting-state effective connectivity of this circuit was modeled by employing spectral dynamic causal modeling in 11 poststroke patients with cognitive impairment (PSCI), 8 poststroke patients without cognitive impairment (non-PSCI) at baseline and 3-month follow-up, and 28 healthy controls. Our results showed that different neuronal models of effective connectivity in the prefrontal–basal ganglia circuit were observed among healthy controls, non-PSCI, and PSCI patients. Additional connected paths (extra paths) appeared in the neuronal models of stroke patients compared with healthy controls. Moreover, changes were detected in the extra paths of non-PSCI between baseline and 3-month follow-up poststroke, indicating reorganization in the ipsilesional hemisphere and suggesting potential compensatory changes in the contralesional hemisphere. Furthermore, the connectivity strengths of the extra paths from the contralesional ventral anterior nucleus of thalamus to caudate correlated significantly with cognitive scores in non-PSCI and PSCI patients. These suggest that the neuronal model of effective connectivity of the prefrontal–basal ganglia circuit may be sensitive to stroke-induced cognitive decline, and it could be a biomarker for poststroke cognitive impairment 3 months poststroke. Importantly, contralesional brain regions may play an important role in functional compensation of cognitive decline.

## Introduction

Most cognitive domains in stroke patients are damaged compared with the non-stroke population (1). Poststroke cognitive impairment often leads to dementia (2) and disability in life (3). Basal ganglia and/or thalamic strokes frequently result in cognitive impairment (4, 5). The pathophysiology underlying cognitive dysfunction is not well understood. Resting-state functional magnetic resonance imaging (rs-fMRI) studies have shown that poststroke cognitive impairment may be associated with specific functional alterations (6) and an abnormal pattern of networks (7), and such alterations may correlate with connectivity changes of the basal ganglia network (8).

Dysfunction of the prefrontal–basal ganglia circuit has been reported in many neuropsychiatric syndromes, including schizophrenia (9) and subcortical ischemic vascular disease (10). Damage to the caudate nucleus (CAU) of basal ganglia and the thalamus can be associated with poststroke cognitive impairment (11). Within the basal ganglia loops of Brodmann area 9 (BA9) of the prefrontal cortex, the activity changes in the caudate globus pallidus internus (GPi) and ventral anterior nucleus of thalamus (VA) is associated with cognitive performance in human (12). The BA9-CAU-GPi-VA-BA9 loop in the prefrontal–basal ganglia circuit may therefore be involved in poststroke cognition and its recovery, a topic not explored previously. Spectral dynamic causal modeling (spDCM) (13) of rs-fMRI data is a technique well-adapted to study this problem, and it has been effectively used to study changes in connectivity associated with impaired consciousness circuit (14, 15) and disrupted sensorimotor circuit (16). spDCM can be used to analyze the effective connectivity, which is defined as the influence one region exerts on another. Analyses of effective connectivity have been used for stroke patients to facilitate motor recovery (17, 18).

The present study therefore applied spDCM on rs-fMRI data to analyze the relationship between effective connectivity of the BA9-CAU-GPi-VA-BA9 circuit and poststroke cognition. We hypothesized that poststroke cognitive impairment may alter the neuronal models of effective connectivity and may be related to the change of specific connectivity paths within the prefrontal–basal ganglia circuit.

## Materials and Methods

### Materials

Forty first-time stroke patients aged 30–60 were recruited for the study from the Department of Neurology, Beijing Tiantan Hospital, Capital Medical University, Beijing, between December 1, 2014 and May 31, 2016. The inclusion criteria were: (A) the lesions of patients were subcortical, i.e., in basal ganglia, thalamus, corona radiata, periventricular white matter, or internal capsule; (B) there was no previous history of stroke or transient ischemic attack; (C) the patient had an informant who knew and had met with the patient on a weekly basis for at least 5 years prior to recruitment. Twenty-nine age-matched healthy controls (HC) participants from the community with no previous history of neurological or psychiatric disease were recruited for the study. All participants underwent cognitive assessment and MRI. We visually evaluated the severity of white matter hyperintensities, lacunes, Virchow-Robin spaces and microbleeds on the MRI scans of all participants. All subjects were right-handed, and all stroke patients had right hemiparesis. The individual diagnostic information can be found in Supplementary Materials.

### Neuropsychological Assessment

All participating patients underwent neuropsychological assessment by a neurologist within 10 days (baseline) and 3 months after stroke. The assessment was performed before the MRI scan. The severity of stroke was evaluated using the National Institute of Health Stroke Scale (NIHSS) (19). Depression was assessed using the Hamilton Depression Rating Score (HAMD) (20). Basic daily functioning was assessed by the Katz basic activities of daily living (ADL) scale (21). The Beijing version of the Montreal Cognitive Assessment (MoCA-Beijing) scale (22) was used to screen the overall cognitive status of participants. Cognitive impairment was identified by a cut-off point of 22/23 on MoCA-Beijing (23). The stroke patients were then divided into two groups at baseline: patients with poststroke cognitive impairment (PSCI, *N* = 23) and patients without poststroke cognitive impairment (non-PSCI, *N* = 17).

### Image Acquisition

Magnetic resonance imaging data were acquired from all participants on a Siemens 3.0 T Prisma MRI scanner (Siemens Healthcare, Erlangen, Germany) at the Functional Neuroimaging Department, Beijing Neurosurgical Institute, Capital Medical University. Resting-state functional images were acquired using an echo-planar-imaging (EPI) sequence with repetition time (TR) = 2,500 ms, echo time (TE) = 30 ms, flip angle = 90°, voxel size = 2.86 × 2.86 × 3 mm^3^, image matrix = 70 × 70 × 43, 200 volumes. A high-resolution structural T1-weighted anatomic sequence was also acquired with the following parameters: TR = 2300 ms, TE = 2.3 ms, flip angle = 8°, voxel size = 0.94 × 0.94 × 1 mm^3^, image matrix = 256 × 256 × 192.

### Image Preprocessing

Neuroimaging data were preprocessed using SPM12 (http://www.fil.ion.ucl.ac.uk/). The initial 10 volumes of each functional dataset were discarded before the slice-timing correction with the new first volume as a reference. The remaining 190 volumes were realigned and corrected for any head motion using rigid body registration; and then normalized into the EPI-template space, followed by 8-mm FWHM smoothing and band-pass filtering (0.01–0.08 Hz).

### Final Sample

We removed participants from the study if they have one of the following conditions: (1) left hemiparesis or left-handed; (2) absent at 3-month follow-up; (3) head motion during the MRI acquisition was >2 mm in translation or 2° in rotation. Twelve PSCI (8 patients with left hemiparesis), nine non-PSCI patients and one healthy control participant were thus removed from our study. Demographical and clinical information of the participants finally included in our study were summarized in Table 1.

**Table 1:** Demographic and clinical data.

**Clinical characteristics**	**HC (*n* = 28)**	**PSCI patients (*n* = 11)**	**Non-PSCI patients (*n* = 8)**	**Statistical test**	***p***
Sex (male: female)	18:10	9:2	7:1	χ^2^ = 2.321	0.313
Age (years)	50.03 ± 8.08	51.72 ± 9.39	45.62 ± 9.86	*F* = 1.198	0.311
Education (years)	10.86 ± 2.08	9.27 ± 1.62	12.25 ± 1.67	*F* = 5.705	0.006
NIHSS	—	3.27 ± 2.37	2.12 ± 1.25	*t* = 1.244	0.230
HAMD	—	2.27 ± 2.10	2.71 ± 2.69	*t* = −0.390	0.701
ADL	—	20.73 ± 1.27	20.00 ± 0.00	*t* = 1.896	0.087
MoCA (baseline)	26.25 ± 2.44	21.45 ± 3.14	25.25 ± 2.87	*F* = 12.642	<0.0001
MoCA (3 months)	26.25 ± 2.44	21.00 ± 3.52	26.87 ± 1.46	*F* = 18.110	<0.0001

*All subjects were right-handed and all stroke patients had right hemiparesis*.

*HC, healthy controls; PSCI, poststroke with cognitive impairment; Non-PSCI, poststroke without cognitive impairment; NIHSS, National Institutes of Health Stroke Severity; HAMD, Hamilton Depression Rating Score; ADL, Activities of Daily Living Scale; MoCA, Montreal Cognitive Assessment. The chi-square was used for testing differences by Sex, one-way ANOVA for testing Age, Education, MoCA at baseline and 3 months follow-up, and two-samples test for NIHSS, HAMD, ADL*.

### Regions of Interest

**Figure 1:**
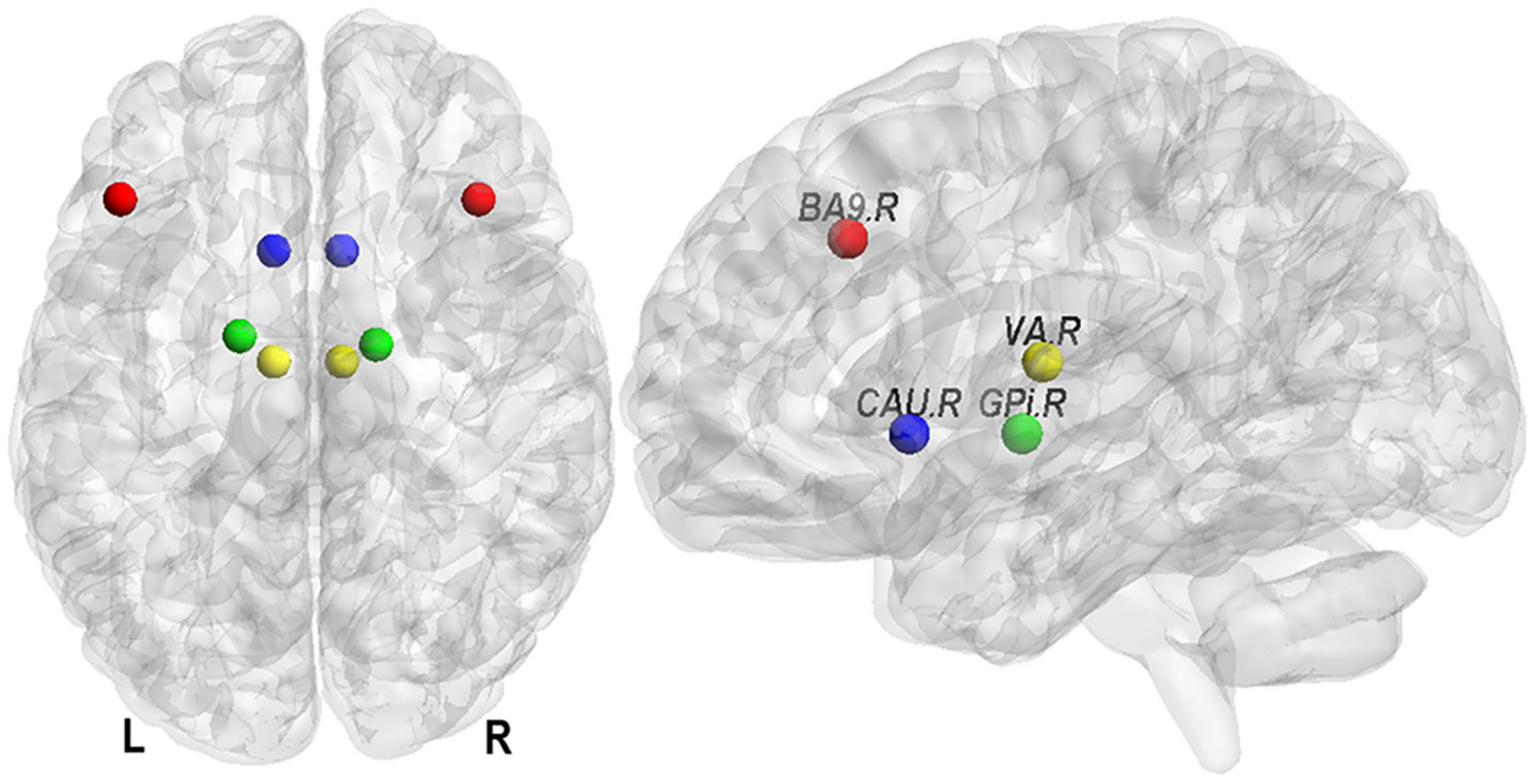
Eight nodes of the prefrontal–basal ganglia circuit. They are Brodmann area 9 (in prefrontal cortex, BA9), caudate nucleus (in basal ganglia, CAU), globus pallidus interna (GPi), and ventral anterior nucleus (in thalamus, VA) in left (L) and right (R) hemisphere.

Previous studies suggested that a stroke event affected not only the lesioned hemisphere but also the contralesional hemisphere (8). Therefore, our prefrontal–basal ganglia circuit included the brain structures in both hemispheres, i.e., left and right CAU (CAU.L, CAU.R) of basal ganglia, left and right globus pallidus interma (GPi.L, GPi.R), left and right ventral anterior nucleus (VA.L, VA.R) in thalamus, and both left and right Brodmann area 9 (BA9.L, BA9.R) in prefrontal cortex (Figure 1). The mask for CAU was selected based on the Automated Anatomical Labeling Atlas (24, 25). The masks for other regions were selected using the Talairach Daemon (26, 27). These masks were then co-registered into the EPI-template to match with the preprocessed functional images. The time-series of ROI were extracted using the principal eigen-variate of time-series of voxels within the masks, and the 6 rigid-body head motion parameters were used as confound regressors. To ensure the co-registration accuracy, the images of each participant with ROI masks were visually inspected. All subjects in this study were right-handed and all stroke patients had right hemiparesis. Therefore, in this study the ipsilesional hemisphere is the left hemisphere and the contralesional hemisphere is the right hemisphere.

### Definition of Models

The effective connectivity is defined as the influence one neural system exerts over another, either at a synaptic or cortical level (28). In other words, the directionality and strength of the effective connectivity mean the propagation way of information and interaction strength between brain areas. In this study, we used the dynamic causal modeling (DCM) (29) method which run over predefined model space to model the effective connectivity.

**Figure 2:**
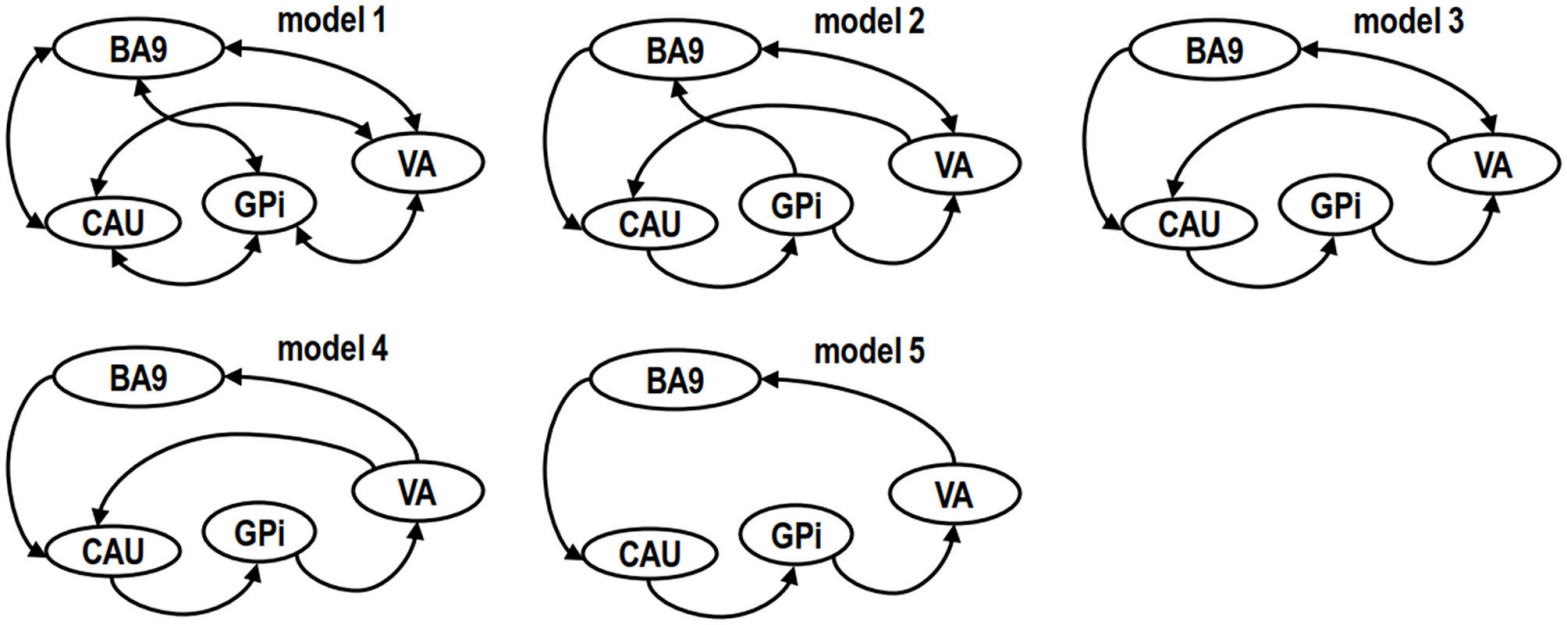
Model spaces of competing hypotheses. Models are specified on the basis of biological knowledge of the cortico–basal ganglia–thalamo–cortical circuit and evidence from other cognitive function studies, targeting central regions or connections in left (L) and right (R) hemispheres on a single-subject basis. The drawing of the models was simplified for readability by representing the same brain structure in both left and right hemispheres with one single node. A unidirectional link between two ROIs represents four paths instead of one. For example, in model 5, the unidirectional link starting from caudate nucleus (CAU) pointing to globus pallidum interna (GPi) has the following 4 paths: (a) CAU.L to GPi.L, (b) CAU.L to GPi.R, (c) CAU.R to GPi.L and (d) CAU.R to GPi.R. Likewise, a bidirectional link between two ROIs represents eight paths instead of just two.

In this work, the definition of model space was hypothesis driven and tested based on the knowledge of empirically verified connectivity patterns between the regions in relation to cognitive function. These regions are known to be anatomically and functionally highly connected and form the cortico–basal ganglia–thalamo–cortical circuit. To test the role of these regions and networks in poststroke cognitive deficits and recovery 3-months after stroke, we created five plausible models in the resting brain (Figure 2). The first was a “full” connection model combining the evidence from previous anatomical and functional studies (30, 31) (Figure 2, model 1).

The second plausible DCM model (Figure 2, model 2) was defined without the backward paths from prefrontal cortex to GPi (BA9 to GPi), thalamus to GPi (VA to GPi), GPi to striatum (GPi to CAU), and forward path from striatum to thalamus (CAU to VA) as suggested by previous studies (14, 32) that proposed that GPi may not directly relay information to cortex. In the third possible model the GPi had no direct path to BA9 (14) (Figure 2, model 3). In the fourth possible model, we assumed an indirect connection from VA to CAU based on a previous study (14) (Figure 2, model 4). In the fifth model, we included only the unidirectional paths, from BA9 to CAU, CAU to GPi, GPi to VA, VA to BA9 (33, 34) (model 5, Figure 2). Other potential models were less plausible than these five models based on Bayesian model selection criteria (35) and were not examined in this study.

### Dynamic Causal Modeling

Spectral DCM (13) was performed using SPM12 on the five models outlined (Figure 2). Briefly, we performed (1) specification of the model space; (2) estimation of the specified models; (3) optimization of the estimated models; (4) implementation of a Bayesian model selection routine to identify the best model for the group based on optimized models; (5) comparison of the best model between the experimental groups [HC, PSCI (baseline, 3 months), non-PSCI (baseline, 3 months)] using the VBA toolbox (36).

The connections between the eight nodes were specified as fixed connections. The connectivity-matrix was specified in the order: CAU.L, CAU.R, GPi.L, GPi.R, VA.L, VA.R, BA9.L, and BA9.R. Each model was fitted with an estimation procedure depending on complex cross spectra over frequencies, i.e., second-order statistics of the cross correlation of the time series. Optimization of DCM was used not only to optimize the effective connectivity strength but also to inspect the correctness of model specification. We employed Bayesian model selection combined with a fixed effect (1st-level, FFX) and random effect analysis (2nd-level, RFX) (35) to identify the best model with the highest posterior evidence. The FFX approach determines which model suits all subjects best multiply the individual Bayes factors. While the RFX approach determines the model with the highest probability of occurrence in the population by investigating how interactions at the neuronal level may have generated the observed data (37). With a combining use of both approaches, we can exclude extreme results that suit one criterion while seriously deviated from another criterion. In this study, the two methods lead to the same results.

### Statistics

The clinical cognitive scores (MoCA) between the groups were compared by conducting a one-way variance analysis (ANOVAs) (*p* < 0.05 false discovery rate (FDR)-corrected (38) for multiple comparisons) between baseline and 3 months. Paired *t*-tests were used to compare the changes of effective connectivity within prefrontal–basal ganglia circuit from poststroke baseline to 3-month follow-up. To explore the relationship between coupling parameters of effective connectivity and cognitive scores, Pearson and Spearman correlation coefficients were computed. The significance threshold was defined at *p* < 0.05 two-tailed. The chi-squared test was used for analyzing differences by sex, one-way ANOVA for testing age, education, MoCA at baseline and 3-month follow-up, and two-sample test for NIHSS, HAMD, and ADL scores.

## Results

### Clinical Data

**Figure 3:**
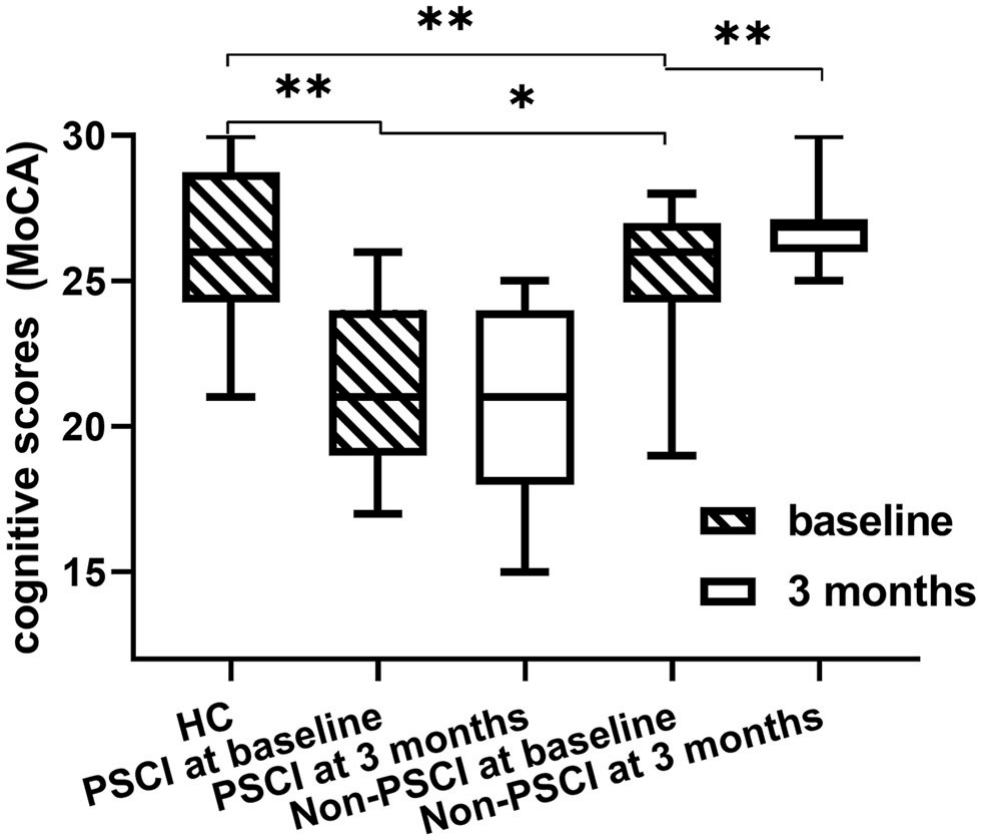
Difference in MoCA scores among healthy controls, PSCI and non-PSCI patients. The MoCA scores of PSCI patients are significantly lower than those of non-PSCI patients and healthy controls. *p < 0.05, **p < 0.001, all the *p*-values were FDR-corrected for multiple comparisons.

The clinical data are presented in Table 1. Comparisons of MoCA scores of all the groups are plotted in Figure 3. The MoCA scores of PSCI patients were significantly lower than those of HC and non-PSCI patients (*p* < 0.0001). The MoCA scores of non-PSCI patients at 3-month follow-up showed a significant recovery in comparison to their baseline. On the other hand, there was no significant difference in PSCI between baseline and 3-month follow-up. While non-PSCI had significantly higher MoCA scores than those of PSCI at baseline, the MoCA scores of both non-PSCI and PSCI at baseline were lower than those of HC.

### Bayesian Model Selection

**Figure 4:**
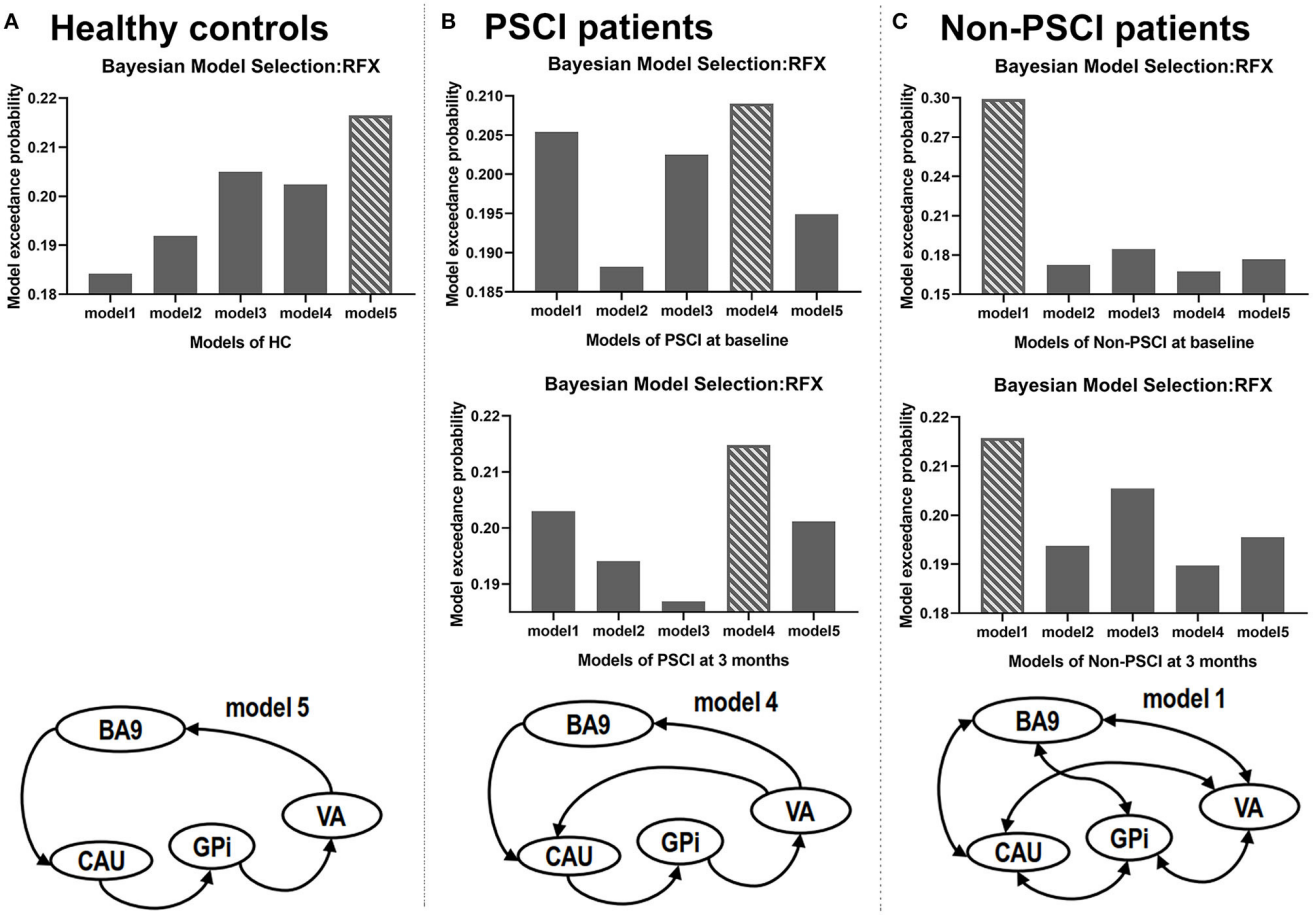
Results of Bayesian model selection. The best model for each sample is shown as the bar with crosslines. RFX, random-effect analysis.

Using Bayesian model selection, five possible alteration neuronal models were compared (Figure 4). The results showed that model 5 (Figure 2) was the best model for healthy controls (Figure 4A), which featured unidirectional paths for BA9 to CAU, CAU to GPi, GPi to VA, VA back to BA9. More connections (extra paths) appeared in the neuronal models of stroke patients. The best model for PSCI patients at both baseline and 3 months was model 4 (Figure 2), which had an extra path from VA directly to CAU comparing with HC (Figure 4B). The best model for non-PSCI patients at both baseline and 3 months was model 1 (Figure 2), which was the fully connected and bidirectional model (Figure 4C).

### Effective Connectivity

The extra effective connections in non-PSCI patients showed significant changes at 3-month follow-up compared to their baseline after stroke. At 3-month follow-up, the connectivity strengths had decreased from GPi.R to CAU.L (*p* = 0.032, *t* = 2.679), from BA9.R to GPi.R (*p* = 0.032, *t* = 2.660), and self-connectivity from VA.L to VA.L (*p* = 0.031, *t* = 2.684), and increased from BA9.L to BA9.R (*p* = 0.011, *t* = −3.400) (*p* < 0.05).

### Correlation With Cognitive Performance

**Figure 5:**
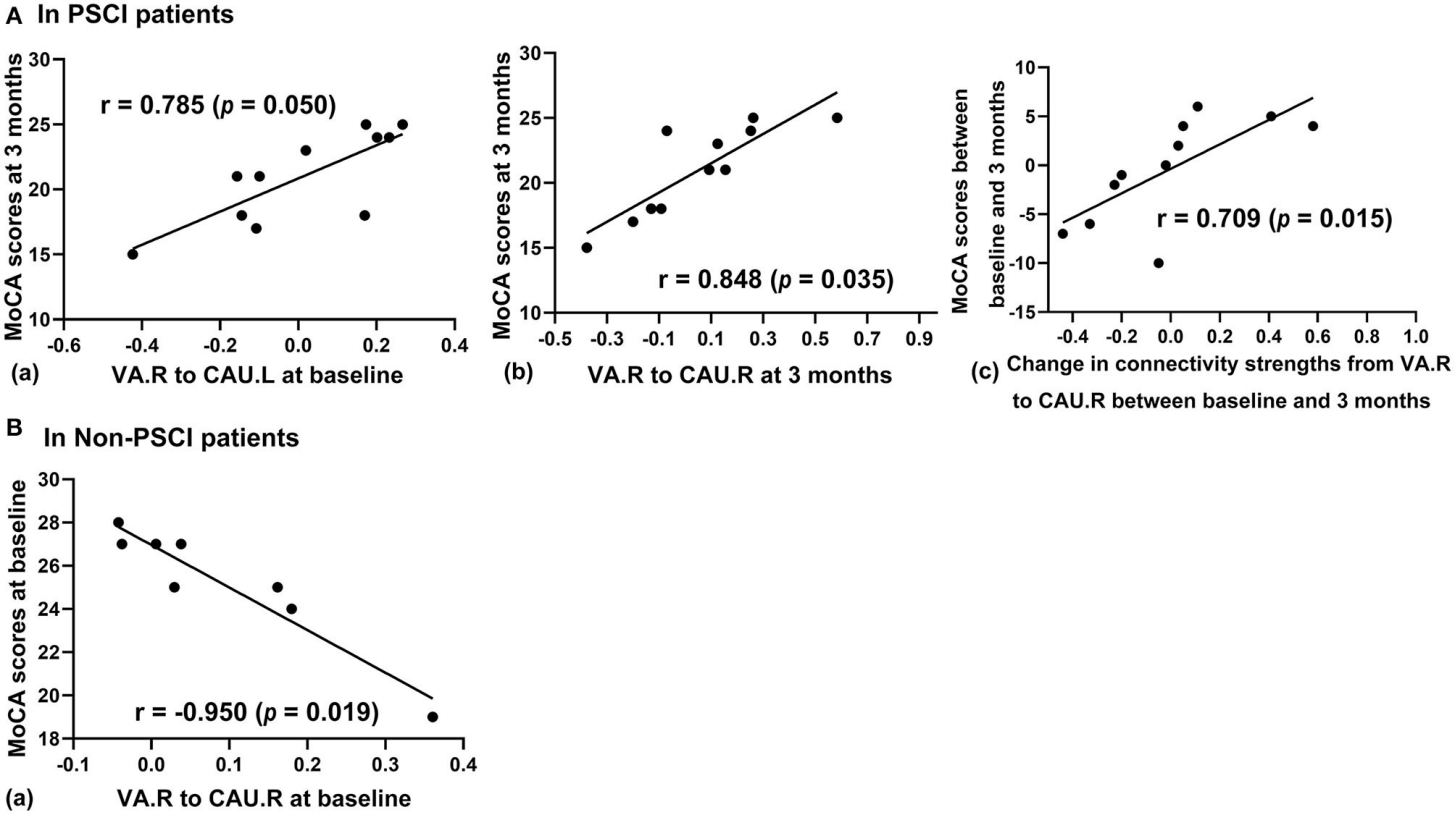
The relationship between MoCA scores and effective connectivity. **(A)** In PSCI patients, (a) cognitive scores at 3 months poststroke correlate significantly with connectivity strengths from VA.R to CAU.L at baseline; (b) cognitive scores correlate significantly with connectivity strength from VA.R to CAU.R at three months; (c) changes of cognitive scores correlate significantly with changes of connectivity from VA.R to CAU.R between baseline and 3 months. **(B)** In non-PSCI patients, cognitive scores negatively correlate with connectivity strengths from VA.R to CAU.R at baseline (*p* < 0.05, FDR-corrected).

The connectivity strengths of extra paths from VA to CAU in PSCI patients significantly correlated with cognition (MoCA scores) by Pearson correlation (Figure 5A). The MoCA scores at 3 months were found to correlate with connectivity strengths from VA.R to CAU.R at baseline (*p*=0.050, *r*=0.786), and connectivity strengths from VA.R to CAU.R at 3 months (*p* = 0.035, *r* = 0.848); the MoCA score changes correlated with the connectivity strength changes from VA.R to CAU.R between baseline and 3 months after stroke (*p* = 0.015, *r* = 0.709). The connectivity strengths of extra connected paths in non-PSCI patients correlated with MoCA scores based on Pearson correlation (Figure 5B). The connectivity strengths from VA.R to CAU.R correlated with MoCA scores at baseline (*p* = 0.020, *r* = −0.950) (*p* < 0.05, all the *p* values were FDR-corrected). These significance correlations were also valid by Spearman correlation (see Supplementary Materials).

## Discussion

This study has advanced our understanding of the changes in effective connectivity in the prefrontal–basal ganglia circuit of poststroke patients in the first 3 months. We found that there were different neuronal network models for healthy controls, non-PSCI and PSCI patients. We also found significant changes between baseline and 3-month follow-up in the extra connected paths of non-PSCI patients, and significant correlations between the extra connections from VA to CAU and cognitive scores in both PSCI and non-PSCI patients. The results indicated that poststroke cognition was associated with alteration of the effective connectivity network in prefrontal–basal ganglia circuit.

One of our main findings was that different groups had different neuronal models of effective connectivity in the prefrontal–basal ganglia circuit. Healthy controls showed unidirectional connected paths, which included the (direct pathways) BA9-CAU, CAU-GPi, GPi-VA, and VA-BA9 in the prefrontal–basal ganglia circuit (Figure 2, model 5). Previous anatomic and physiologic studies in healthy primates showed that the neural circuit formed by these direct pathways may be involved in cognitive activity (39). In contrast, our study found that non-PSCI patients showed fully and bidirectionally connected paths for both baseline and 3-month follow-up after stroke (Figure 2, model 1). Consistently, previous studies also found a fully connected effective-connectivity model of motor network for the patients with poststroke motor impairment and recovery over time (40). Compared with healthy controls, the extra paths in non-PSCI patients may play important roles in reorganization of the neuronal network to bypass deficits due to stroke. On the other hand, the PSCI patients had only one extra path from VA to CAU (Figure 2, model 4) in comparison to the healthy controls, which differed from the fully connected model structure in non-PSCI patients. It was reported that poststroke cognitive impairment was associated with impaired brain network (6), and there was limited compensatory mechanism as demonstrated in the network connectivity patterns (41). Our study found that despite the fact that both PSCI and non-PSCI patients had the extra path from VA to CAU, the PSCI patients had fewer connected paths in the circuit compared to non-PSCI patients and many of them were unidirectional. The absence of extra pathways of the prefrontal–basal ganglia circuit may explain the absence of poststroke cognitive recovery in PSCI patients.

In our study, the longitudinal changes of effective connectivity strength were only observed in non-PSCI patients for the network of the prefrontal–basal ganglia circuit. The results demonstrated that non-PSCI patients' cognitive scores increased 3 months after stroke, with changes to the excitatory connectivity from ipsilesional BA9 to contralesional BA9 and inhibitory self-connectivity of ipsilesional VA. These results suggest that the enhancement of the connections may contribute to ipsilesional neuronal reorganization, which is an important process in cognitive repairment. Previous investigations suggested functional reorganization of cortical network in ipsilesional hemisphere after stroke (42–44). The prefrontal cortex information input and thalamus gate of the prefrontal–basal ganglia circuits (39) can play an important role for poststroke ipsilesional reorganization. In addition, our results also showed that the non-PSCI patients' excitatory connections from contralesional BA9 to GPi, and contralesional GPi to ipsilesional CAU, decreased during the 3 months after stroke. The globus pallidus-frontal cortex cells was reported to comprise a direct GABAergic/cholinergic projection under the control of basal ganglia (32). Findings from other stroke studies suggested that the modulating activity of CAU in cortico–basal ganglia circuits (45) as well as deep brain stimulation of the globus pallidis (46) improved poststroke deficits. These results indicate that the connections may support reorganization in the contralesional hemisphere, which may contribute to the functional compensation of deficits due to stroke. This contralesional effect was reported in the studies for poststroke motor impairment (43, 47).

The relationship between cognitive performance and connectivity strength of the extra path from VA to CAU was found in both non-PSCI and PSCI patients. Our results showed connectivity strength from contralesional VA to CAU at 3 months negatively correlated with cognitive performance at baseline in non-PSCI patients. This indicates that the reduction of contralesional connectivity may contribute to cognitive improvement, which further suggests functional compensation of contralesional hemisphere for poststroke deficits. Similarly, the connectivity strengths from contralesional VA to CAU was also observed to be related with cognitive performance in PSCI patients. The changes of its connectivity strength correlated positively with increased in cognitive scores between baseline and 3-month follow-up in PSCI patients. In addition, the PSCI patients' connectivity strengths from contralesional VA to ipsilesional CAU were positively correlated with improved cognitive performance. These results suggest the enhancement of excitatory connectivity from contralesional VA to CAU at 3-months after stroke may promote cognitive recovery. In a study of patients with thalamic lesions, deficits in cognitive functions were found to be associated with the ventral anterior portion of the thalamus (46), a relay station for information between networks (48). While a study focused on the caudate, which serves as an important center of integration of networks (49), found its lesions in stroke patients are associated with cognitive impairment at short-term (3–6 months) follow-up (11). Our results demonstrated the reduction of excitatory connectivity in contralesional VA to CAU was associated with the decline of cognitive scores in PSCI patients 3 months after stroke. This suggests that only one extra connected path from VA to CAU may be inadequate for supporting effective recovery in poststroke cognitive impairment.

The main limitations of our study are the relatively small subject sample size and the limited number of network nodes of the cortico–basal ganglia circuit we considered for our models. Despite this, the relationship between effective connectivity and MoCA scores can be clearly seen from Figure 5 and the trend is obvious. The circuit can be analyzed with more than eight nodes in studies of functional activity. However, for a study employing the DCM method, it would be difficult to include all the possible nodes and pathways due to the computational complexity (50). Another limitation to note is that we did not mark the lesions for each patient since the resolution of DTI data used for diagnosis was low and partially missing. Therefore, the lesion areas were not specially considered in the processing. In addition, although the years of education of PSCI patients were significantly lower than non-PSCI patients (*p* = 0.005), there was no significant difference (*p* = 0.891) in the test of the selection of optimal models, when the years of education was controlled as the covariate in the univariate analysis of variance. And also, those with PSCI do appear about 5–6 years older than non-PSCI patients, although age is not statistically significant.

## Conclusion

The present study demonstrated the existence of different neuronal models of effective connectivity of prefrontal–basal ganglia circuit among healthy controls, and poststroke patients with and without cognitive impairment. Compared with healthy controls, more connected paths were shown in neuronal models of stroke patients. These extra connected paths may relate with cognitive impairments and recovery after stroke. Furthermore, poststroke cognitive impairment may also interfere the connected paths involved in reorganization of prefrontal–basal ganglia network. Although our findings need further confirmation, they provide us with a better understanding of the pathophysiology of poststroke cognitive impairment and a possible neuroimaging biomarker for future interventional studies.

## Data Availability Statement

The datasets presented in this article are not readily available because the dataset used and analyzed are available to other researchers subject to review of the request by the Scientific Committee of the study and ethics approval. Requests to access the datasets should be directed to tao.liu@buaa.edu.cn.

## Ethics Statement

The studies involving human participants were reviewed and approved by Beijing Tiantan Hospital Ethics Review Board. The patients/participants provided their written informed consent to participate in this study.

## Author Contributions

This writing of manuscript, analysis and interpretation of data were completed by JZ, ZL, and XC. The paper was then amended and approved by TL, YW, WW, WZ, JJ, JC, and PS. The experiment design and data collection were performed by LZ.

## Conflict of Interest

The authors declare that the research was conducted in the absence of any commercial or financial relationships that could be construed as a potential conflict of interest.

## Abbreviations

HC: healthy controls
PSCI: poststroke with cognitive impairment
non-PSCI: poststroke without cognitive impairment
NIHSS: National Institutes of Health Stroke Severity
HAMD: Hamilton Depression Rating Score
ADL: Activities of Daily Living Scale
MoCA: Montreal Cognitive Assessment
rs-fMRI: resting-state functional magnetic resonance imaging
CAU: caudate nucleus of basal ganglia
GPi: globus pallidus internus
spDCM: spectral dynamic causal modeling
DCM: dynamic causal modeling
VA: ventral anterior nucleus of thalamus
BA9: Brodmann area 9
EPI: echo-planar-imaging
TR: repetition time
TE: echo time.
