# Supplementary material for: Altered Prefrontal–Basal Ganglia Effective Connectivity in Patients With Poststroke Cognitive Impairment
Source: Front Neurol. 2020 Dec 16;11:577482. doi: 10.3389/fneur.2020.577482 (PMC7772311; doi:10.3389/fneur.2020.577482)
Supplement: Supplementary file 1 [file Table_1.DOC]

**SupplementaryMaterial_01_lesions**

**Supplementary data**

| PSCI No. | Lesion site | Non-PSCI no. | Lesion site |
| --- | --- | --- | --- |
| 02 | L BG,CR | 01 | L thalamus |
| 04 | L BG,CR | 04 | / |
| 05 | LR BG,CR | 06 | L BG, IC, thalamus |
| 06 | L BG,CR | 07 | L BG,CR |
| 07 | L CR thalamus，R BG | 09 | L CR, IC, R thalamus |
| 12 | R BG,CR | 10 | / |
| 14 | L BG,CR | 11 | L BG,PV |
| 16 | L thalamus | 14 | L BG,CR |
| 17 | R BG,PV |  |  |
| 18 | L BG,CR |  |  |
| 19 | L CR |  |  |

**STable 1:** Lesions in stroke patients

The anatomical locations of stroke lesions of eleven stroke patients with poststroke cognitive impairment (PSCI) and eight stroke patients without cognitive impairment (Non-PSCI). All patients were right hemiplegia. L, left; R, right; LR, left and right; BG, basal ganglia; CR, corona radiate; PV, periventricular; IC, internal capsule.

**SupplementaryMaterial_02_** **PearsonCoefficients**

**Table. Results of effective connectivity strengths and MoCA scores for Spearman correlation.**

| PSCI | Variate | Time | P Value | correlation coefficient |
| --- | --- | --- | --- | --- |
|  | VA.R to CAU.L | baseline | *p*=0.002 | *r*=0.821 |
|  | MoCA | 3 months |  |  |
|  | VA.R to CAU.R | 3 months | *p*<0.001 | *r*=0.908 |
|  | MoCA | 3 months |  |  |
|  | *d*VA.R to CAU.R | Between baseline and 3 months | *p*=0.001 | *r*=0.861 |
|  | *d*MoCA |  |  |  |
| Non-PSCI | VA.R to CAU.R | baseline | *p*=0.002 | *r=-0.908* |
|  | MoCA | baseline |  |  |

*PSCI, poststroke with cognitive impairment; Non-PSCI, poststroke without cognitive impairment; MoCA, Montreal Cognitive Assessment; L, left; R, right; d, changes between baseline and 3 months follw-up.*
